# Abandoned Cataract Surgeries: An Analysis of Surgical Referrals to a Tertiary Eye Center

**DOI:** 10.7759/cureus.74457

**Published:** 2024-11-25

**Authors:** Aafreen Bari, Tushar Agarwal, Tanuj Dada, Namrata Sharma

**Affiliations:** 1 Ophthalmology, All India Institute of Medical Sciences, New Delhi, IND

**Keywords:** abandoned cataract surgery, blindness, management, ocular emergency, visual impairment

## Abstract

Purpose: This study aims to analyze the outcomes of cases of abandoned cataract surgeries referred to a tertiary eye center.

Methods: This retrospective observational case series includes eleven cases referred to a tertiary eye center following abandoned cataract surgeries. The preoperative factors, intraoperative management, and postoperative outcomes were recorded and analyzed.

Results: The mean age of presentation was 56.8 ± 13.7 years, and the median time from the primary surgery to presentation at the tertiary eye center was four days (range: two hours to four weeks). There was an association with hypermature cataracts in 36.3% (4/11) of cases, diabetes in 36.3% (4/11) of cases, and pseudo-exfoliation syndrome in 18.2% (2/11) of cases. The primary cataract surgery was aborted after making an incision and without initiating capsulorhexis in 45.5% (5/11) of cases. Descemet's membrane detachment and corneal edema were present in nearly half (45.5%) of the cases.

Conclusion: A thorough preoperative assessment, early identification of intraoperative complications, timely referral by the primary surgeon, and appropriate and prompt intervention by the second surgeon are essential for improving visual outcomes.

## Introduction

Cataract surgery and the teaching of this art to future generations of ophthalmologists have evolved significantly over the decades. The techniques for nucleus extraction have transformed from extracapsular cataract extraction to small-incision cataract surgery and, most recently, to minimally invasive small-incision phacoemulsification. Globally, approximately 232,866 ophthalmic surgeons are treating various ocular conditions. On average, low-income countries have 3.7 ophthalmologists per million population, compared to 76.2 in high-income countries [1].

With increasing life expectancy and population growth, cataracts remain the leading cause of blindness worldwide [2]. Unfortunately, iatrogenic complications resulting from cataract surgery continue to be a significant cause of blindness. According to the National Blindness and Visual Impairment Survey of India (NPCB report 2015-2019, conducted by the Rajendra Prasad Centre for Ophthalmic Sciences, New Delhi), complications following cataract surgery accounted for 7.2% of total blindness, making it the third most common cause [3]. Cataract surgeries terminated at any intraoperative step by the surgeon are defined as abandoned cataract surgeries, which can critically contribute to vision loss following cataract surgery.

When surgeons encounter an accidental mishap during cataract surgery, early identification is crucial for minimizing further damage. Various factors that can help ophthalmic surgeons optimize visual outcomes in phacoemulsification include proper instillation of peribulbar anesthesia, appropriate sizing and placement of incisions, a suitable nucleotomy technique, identification and management of posterior capsular tears, intraocular lens (IOL) injection, and wound closure [4]. A recent review by Goel et al. demonstrated that while manual small-incision cataract surgery is universally applicable and yields consistent visual outcomes, it poses a significant learning curve [5].

There is an extensive body of research highlighting cataracts as the leading cause of blindness and discussing effective extraction techniques [6]. However, there is a notable lack of literature on cases where surgeries are abandoned by the primary surgeon and referred to higher centers for further management. This study aims to analyze a series of cataract cases that were aborted intraoperatively and referred to a tertiary eye center for further care.

## Materials and methods

This retrospective observational case series included cases presenting to the eye emergency department with a history of abandoned cataract surgery. The study was conducted at the Dr. Rajendra Prasad Centre for Ophthalmic Sciences, All India Institute of Medical Sciences, Delhi, from December 2023 to May 2024. Ethical clearance was obtained from the Institutional Ethical Committee of All India Institute of Medical Sciences (approval number: AIIMSA2246). The study adhered to the tenets of the Declaration of Helsinki.

All cases aged over 18 years who had undergone previously aborted cataract surgery and were referred to a tertiary eye center for further management were included. Patients with incomplete data were excluded from the study. All cases who consented to undergo a second surgery for subsequent management were included.

Preoperative findings at presentation, including visual acuity, intraocular pressure (IOP), slit lamp examination with clinical photography, fundus examination, and ultrasonography B-scan, were documented. Intraoperative findings and surgical videos were assessed. Postoperative data, including visual acuity, IOP, slit lamp examination with clinical photography, and fundus examination, were documented on postoperative day 1 and at six weeks. All data were entered into an Excel sheet (Microsoft Corporation, Redmond, WA, USA) and analyzed.

## Results

The series includes 11 cases that were referred to or presented at our tertiary eye center shortly after undergoing incomplete cataract surgery. The average age of patients in the study was 56.8 ± 13.7 years. The median time from primary surgery to presentation at the tertiary eye center was four days (interquartile range: 11.5). An association with diabetes was observed in 36.3% (4/11) of cases, and pseudo-exfoliation syndrome was present in 18.2% (2/11) of cases. In 45.5% (5/11) of cases, the surgeons abandoned cataract surgery after making the incision without initiating capsulorhexis. Descemet’s membrane detachment (DMD) and corneal edema were noted in 45.5% (5/11) of cases. Additionally, hypermature cataract was associated with 36.3% (4/11) of cases. There were no instances of postoperative infection. The details of each case have been discussed individually.

Cases 1 and 2

**Figure 1:**
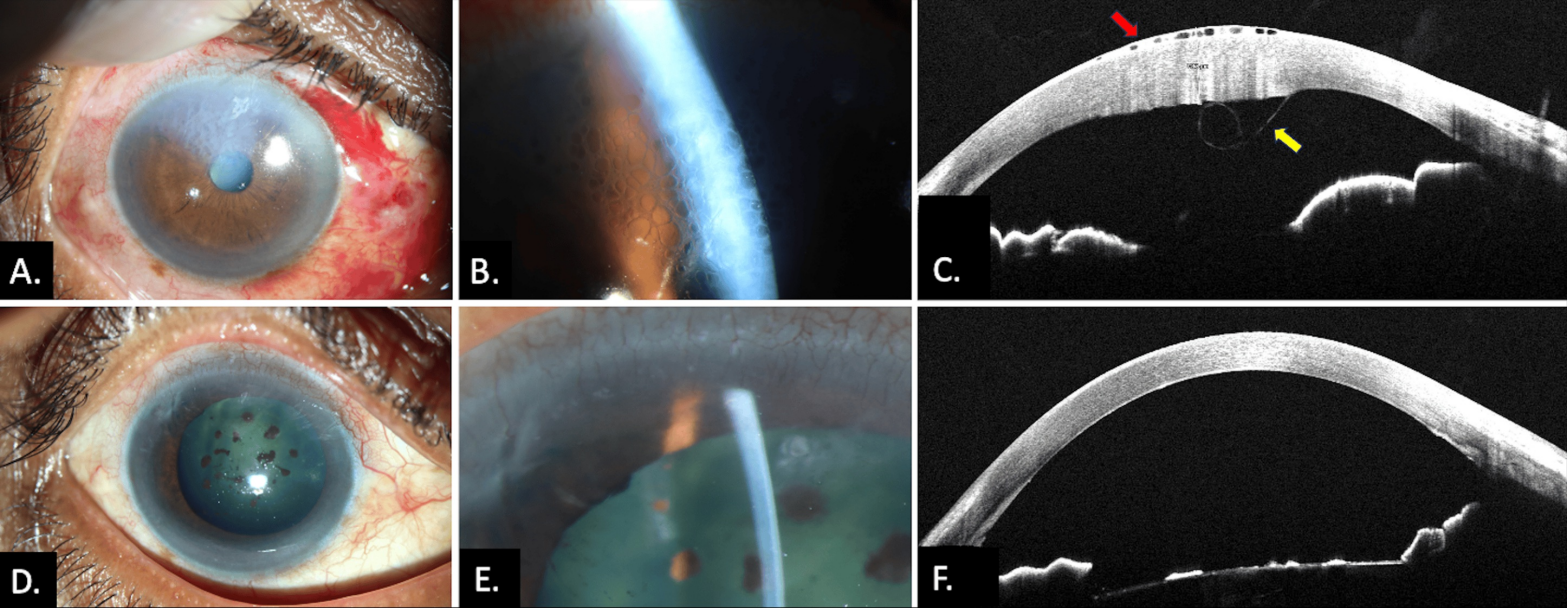
Clinical photograph of Case 2 showing the right eye with (A) corneal edema in the superior quadrant. (B) Typical honeycomb-shaped reticular epitheliopathy. (C) AS-OCT shows honeycomb-shaped epithelial edema (red arrow), stromal edema, and scrolled Descemet’s membrane (yellow arrow). (D) Postoperative image with resolved corneal edema. (E) Re-attached Descemet’s membrane with compact cornea. (F) AS-OCT shows postoperative compact corneal stroma with normal epithelium and reattached Descemet’s membrane AS-OCT: anterior segment optical coherence tomography

Case 1 involved a 53-year-old female who presented with watering and photophobia in the left eye (LE). She had undergone phacoemulsification with IOL implantation in the LE two days prior at another facility. On examination, central DMD with corneal edema was noted. The anterior segment optical coherence tomography (AS-OCT) confirmed the findings. Case 2 involved a 54-year-old male with similar complaints, presenting one month after an abandoned cataract surgery. He had a superior scleral incision, DMD, and corneal stromal edema with honeycomb epitheliopathy. After thorough counseling and obtaining informed consent, both patients underwent descemetopexy with intracameral air injection (Video 1). On follow-up, the DMD and epitheliopathy resolved, with improvement in visual acuity (Figure 1).

**Video 1:** Video showing the real-time assessment of the reattachment of DMD as appreciated on Mi-OCT Mi-OCT: microscope-integrated optical coherence tomography, DMD: Descemet’s membrane detachment

Case 3

**Figure 2:**
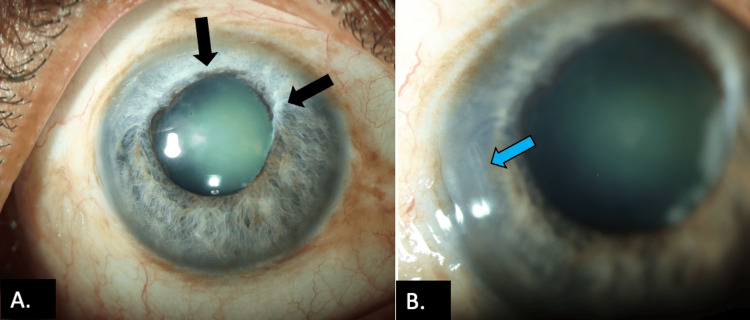
Clinical photograph of Case 3 showing (A) hypochromic iris, iridoschisis, pupillary ruff atrophy, and irregular-shaped pupil (black arrows) and (B) temporal incision tract scar (blue arrow) of previous entry

A 47-year-old man presented with blurred visual acuity in the right eye (RE). He had a history of RE cataract surgery that was aborted intraoperatively a month earlier. On examination, the patient had a slightly eccentric, poorly dilating (4 mm) pupil with pupillary ruff atrophy, heterochromia, and iridoschisis (Figure 2A). There was a temporal clear corneal scar, suggesting the entry point of the abandoned cataract surgery (Figure 2B). After thorough ocular investigations, the patient underwent iris hook-assisted phacoemulsification with IOL implantation in the same eye. A second surgeon performed the procedure using an alternate (superior) incision site.

Cases 4 and 5

Case 4 was an 87-year-old male patient referred one day after an abandoned cataract surgery. He had pseudo-exfoliation syndrome with grade IV nuclear sclerosis and subluxation. On presentation, 24 hours after his primary surgery, he exhibited pseudo-exfoliation syndrome, a hypermature cataract, and subluxation. The primary surgeon had made a sealed corneal incision. Due to subluxation involving 8-9 clock hours, the second surgeon performed intracapsular cataract extraction with anterior chamber IOL implantation. On follow-up, the patient had a stable IOL with a clear cornea.

Case 5 was a 55-year-old male who presented seven days after an abandoned cataract surgery. The primary surgeon had documented intraoperative fluid misdirection with high IOP. On presentation, the patient had a superiorly constructed wound closed with a suture and the iris plastered to the inner lip of the wound. His IOP was 20 mmHg while on dual topical antiglaucoma therapy (brimonidine 0.5% + timolol 0.5%) and oral acetazolamide (250 mg three times per day). He was initially managed medically to control the IOP. Once the IOP was stabilized, he underwent phacoemulsification with IOL implantation under acetazolamide cover. A temporal incision was made to avoid the area of anterior synechiae. Postoperatively, the patient achieved an uncorrected distance visual acuity of logMAR 0.18, and his IOP was 14 mmHg, well-controlled with a single antiglaucoma medication (timolol 0.5% eye drops).

Cases 6 and 7

**Figure 3:**
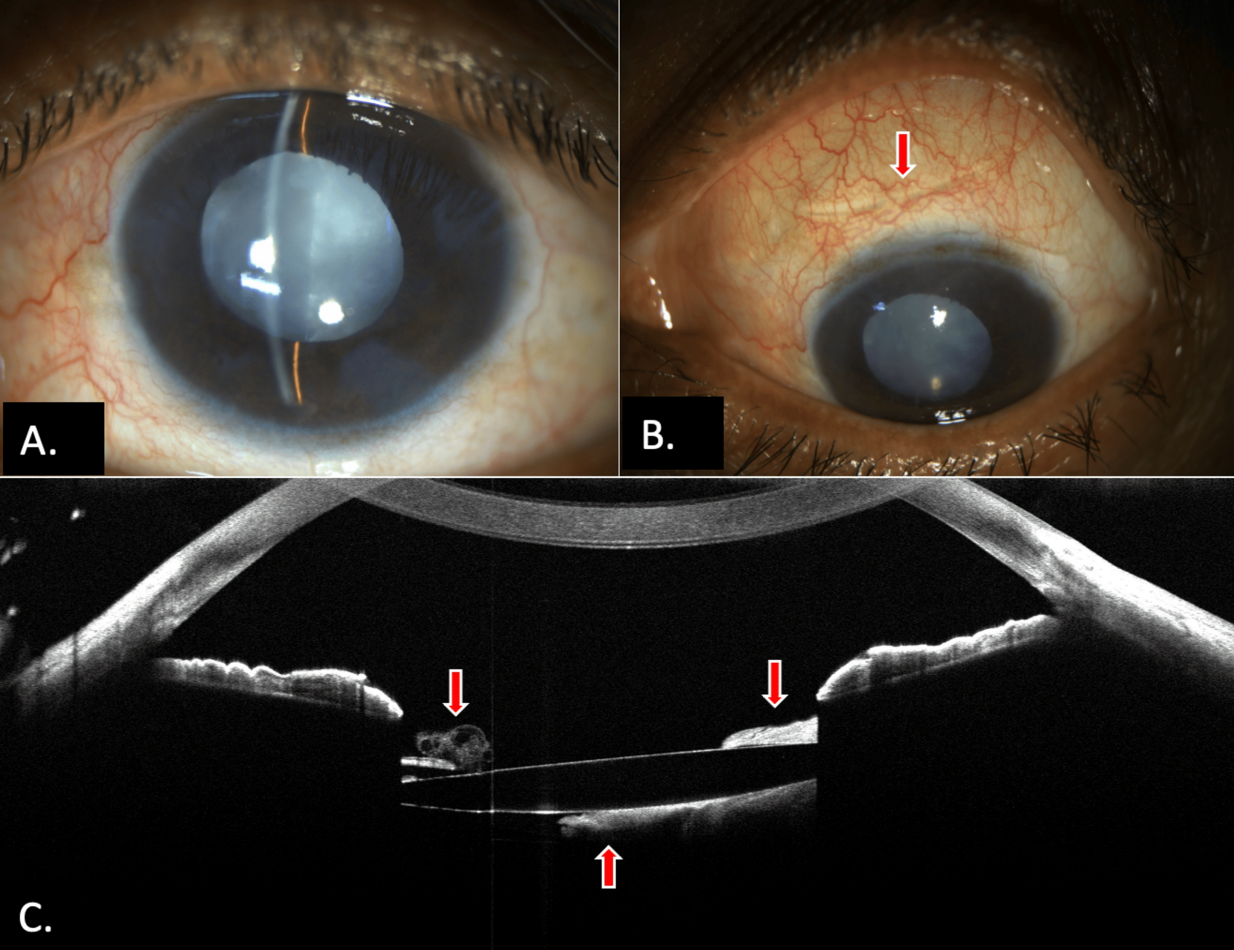
Clinical photograph of Case 6 showing (A) clear cornea with IOL in situ with a white membrane-like curtain behind IOL, (B) superior scleral scar of previous entry (red arrow), and (C) AS-OCT showing retained lens and cortical matter (red arrows) with IOL in situ IOL: intraocular lens, AS-OCT: anterior segment optical coherence tomography

Two patients (Case 6 and Case 7) presented with unsatisfactory visual acuity two weeks following phacoemulsification and IOL implantation. The visual acuity of Case 6 was limited to hand movements close to the face, with a completely retained sheet of cortical matter behind the IOL (Figure 3). In contrast, Case 7 had a visual acuity of 20/63 with a partially retained cortical sheet, as documented on AS-OCT. Both patients underwent cortical matter removal using bimanual irrigation and aspiration, resulting in improved visual acuity of 20/40 and 20/32 in Case 6 and Case 7, respectively.

Case 8

A 55-year-old male was referred from a local hospital with a case of RE hazy cornea and difficult intraoperative management. He presented with photophobia, watering of the RE, and a history of RE phacoemulsification with IOL implantation six hours earlier. On examination, the patient's visual acuity was limited to hand movements close to the face, and IOP was 5 mmHg (measured using non-contact tonometry). A large (approximately four millimeters) temporal corneal incision, which tested Seidel-positive on fluorescein staining, was noted.

After obtaining written informed consent from the patient, the wound integrity was secured under topical anesthesia using a single 10-0 nylon suture. During the injection of balanced salt solution into the anterior chamber, Descemet’s membrane scroll slipped out through the remaining portion of the incision. It was carefully repositioned through the same incision. Using microscope-integrated optical coherence tomography, the configuration of Descemet’s membrane scroll was evaluated. Since it was found to be in the correct orientation, an Auto-DMEK (Descemet’s membrane endothelial keratoplasty) procedure was attempted. Intracameral air was injected, ensuring optimal adhesion at the graft-host junction (Video 2). After a few minutes, the anterior chamber was decompressed, and the patient was monitored. At the four-week follow-up, the corneal edema had resolved completely. The patient’s vision improved to 20/20 with no evidence of DMD.

**Video 2:** Video showing the Auto-DMEK technique in a case of scrolled DMD Auto-DMEK: Descemet’s membrane endothelial keratoplasty, DMD: Descemet’s membrane detachment

Cases 9 and 10

Case 9 involved a 74-year-old female referred to the tertiary eye center following an abandoned cataract surgery for a diabetic hypermature cataract. She presented with aphakia, a wound leak from the small incision cataract surgery (SICS) tunnel, DMD, corneal edema, and vitreous in the anterior chamber touching the endothelium. She was counseled about the risks of endophthalmitis, corneal decompensation, and poor visual outcomes. She underwent anterior vitrectomy, intrascleral fixation of a three-piece IOL, SICS tunnel closure, and descemetopexy (Video 3). At the three-week follow-up, she achieved a visual acuity of 20/80 with an IOP of 14 mmHg. She was also counseled about the potential need for corneal transplantation in the future.

**Video 3:** Video showing an abandoned cataract surgery with aphakia and open scleral wound undergoing wound closure and intra-scleral haptic fixation of a three-piece IOL IOL: intraocular lens

Case 10 was a similar case. He was a 58-year-old male referred following an abandoned SICS for a subluxated hypermature cataract. The patient was aphakic and had vitreous prolapse through the SICS tunnel. He was counseled about the risks of infection and other ocular complications. He underwent wound-site vitrectomy, anterior vitrectomy, and intra-scleral haptic fixation of a three-piece IOL. On follow-up, his visual acuity improved to 20/40.

Case 11

**Figure 4:**
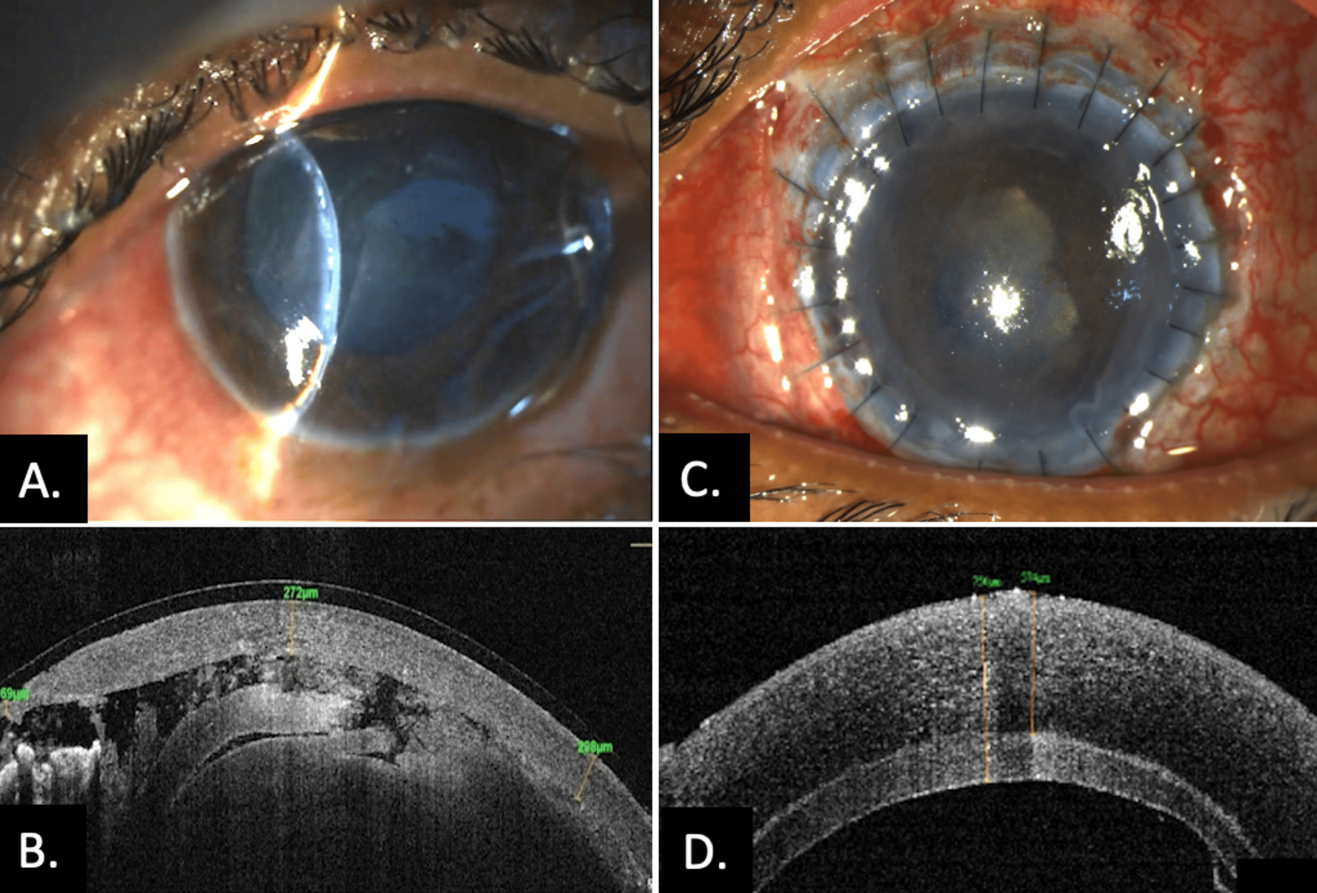
Preoperative clinical photograph of Case 11 showing (A) diffuse corneal haze with flat anterior chamber and lens-endothelial touch. (B) AS-OCT image showing corneal edema with lens-endothelial touch. (C) Postoperative clinical image after deep anterior lamellar keratoplasty with formed anterior chamber and intact sutures. (D) Postoperative AS-OCT with well-apposed graft-host junction AS-OCT: anterior segment optical coherence tomography

A 30-year-old male with a known case of corneal ectasia in both eyes was referred from a local eye hospital with a spontaneous corneal rupture in his LE following trivial trauma. He underwent an attempt at glue-based bandage contact lens with lens aspiration, but the surgery was aborted due to a failed attempt to close the primary wound. His RE was phthisical following trauma sustained 10 years ago. Upon presentation, the visual acuity in the LE was perception of light with accurate projection of rays. Slit-lamp examination of the LE revealed diffuse corneal edema, a temporal limbal corneal perforation, a flat anterior chamber, and an intumescent cataractous lens touching the corneal endothelium (Figure 4A). Ultrasonography of the LE showed an anechoic signal, and AS-OCT indicated a central corneal thickness of 272 μm (Figure 4B). The systemic examination was normal. A diagnosis of brittle cornea syndrome was made based on the history of perforation following trivial trauma and the abnormal integrity of the wound that could not be closed by the primary surgeon. The patient underwent tectonic epi-keratoplasty and lens aspiration with posterior chamber IOL implantation in the LE. On the first postoperative day, the anterior chamber was formed, and the graft was in situ with all sutures intact (Figure 4C). AS-OCT showed a well-apposed donor-host junction (Figure 4D). The postoperative visual acuity of the LE was counting fingers close to face with accurate projection of rays, and the IOP was 12 mm Hg.

## Discussion

The incidence of abandoned cataract surgeries and the percentage of cases resulting in visual impairment or blindness cannot be commented upon due to the minimal to poor reportability of these cases. There is little to no literature on instances of aborted cataract surgeries. This study aims to discuss various cases of abandoned cataract surgeries, the steps by which they most commonly occur, the possible reasons for abandonment, and management solutions for these cases.

Cataract surgeons may abandon surgeries at any given step, starting from the entry incision, rhexis, nucleotomy, and final wound closure. There may be certain acceptable reasons for stopping a surgery, which may prevent a general ophthalmologist from proceeding and potentially cause further damage to already compromised ocular microstructures. In this study, cataract surgery was abandoned in 45.5% of cases before the initiation of capsulorhexis. The likely reasons for abandoning cataract surgery at this stage include a sudden rise in IOP due to aqueous misdirection, other ocular pathologies, or poor visibility caused by corneal haze. In cases involving complications that require a targeted approach, such as DMD or nucleus drop, surgeons with specialized training are better equipped to manage these cases, thereby improving surgical outcomes.

Conversely, it may not be justifiable to abandon cataract surgery due to preoperative factors such as the hazy or ectatic cornea, shallow anterior chamber, advanced cataract grade, preoperative zonular dialysis, and pre-existing posterior capsule defect, as these indicate a poor preoperative assessment.

In this study, no significant correlation was observed between systemic comorbidities, eye pathologies, and the rate of abandoned cataract surgeries in these cases. In the review by Hanna et al., it was observed that routine preoperative evaluation did not demonstrate a statistically significant impact on the cumulative incidence of systemic complications, including hypertension, bronchospasm, arrhythmia, and major adverse cardiovascular events, or ophthalmic complications such as posterior capsular rupture and endophthalmitis. However, a notably lower risk of complications was observed among high-risk patients who underwent a comprehensive preoperative evaluation. Although patients with comorbidities are generally at higher risk for adverse events, the precise impact of preoperative assessment in this population remains unclear. The authors emphasized that a thorough preoperative evaluation could potentially reduce the likelihood of intraoperative complications [7,8].

A thorough preoperative evaluation is essential for planning the surgery. A case of pseudo-exfoliation syndrome has up to 25 times higher chances of posterior capsular rent and 37 times higher chances of zonular dialysis compared to routine cases, as assessed by a meta-analysis by Vazquez-Ferreiro et al. [9,10]. It was assumed that other factors, such as variations in nucleotomy techniques, machine principles and probe sizes, type of anesthesia, type of pupil expanders in small pupils, duration of surgery, and surgeon experience, may also contribute to these risks. However, since there were innumerable variations in association, these factors were not assessed. A review by Qureshi and Steel showed that the risk of pseudophakic retinal detachment is 10 times higher than in the general population. The risk factors related to this were intraoperative factors (operative complications, surgeon grade, subsequent laser capsulotomy), intrinsic eye-related factors (laterality, myopia, previous rhegmatogenous retinal detachment, previous trauma, previous posterior vitreous detachment), and patient factors (sex, age, ethnicity, affluence, systemic comorbidities) [11]. Among these, only intraoperative factors were modifiable to decrease the chances of retinal detachment and improve visual outcomes.

Similarly, Sharma et al. described that one of the major factors for corneal edema is DMD. The incidence of unobvious DMD in seemingly uncomplicated cataract surgery may be as high as 47%. The risk factors can be divided into pre-, intra-, and postoperative categories. Preoperative causes include pre-existing endothelial dysfunction, age >65 years, and dense cataracts. Intraoperative risk factors include the use of blunt instruments, inadvertent insertion of instruments between the corneal stroma and Descemet’s membrane, inappropriate incisions, tight main incisions, damage to Descemet’s membrane during irrigation/aspiration, IOL or phacoemulsification probe insertion, and surgeon inexperience [12]. In cases of corneal opacities with cataracts, Sharma et al. described that optimum case selection, choosing the safe technique, and having a thorough understanding of the difficulties and methods for addressing such cases are paramount in achieving optimal visual outcomes [13]. A tailored approach based on the case should be adopted to improve outcomes. For instance, cases with cataracts and microcornea are best approached through a scleral tunnel, which decreases the chances of DMD [14].

Posterior capsule rupture can occur both among experienced senior surgeons and less experienced surgeons, although it is more frequent among the latter group. Additionally, certain cataract compositions are more prone to this complication. If addressed properly and in a timely manner, the eventual outcome may be no different than that of an uncomplicated case. However, improper handling could lead to serious complications with an increased incidence of permanent visual impairment [15].

In our study, certain factors such as advanced cataract grade, pseudo-exfoliation syndrome, subluxated lens, and brittle cornea syndrome could have been diagnosed by the primary surgeon preoperatively. A meticulous preoperative examination might have helped them predict intraoperative challenges, allowing for better preparation for the primary surgery rather than abandoning the procedure at any stage intraoperatively.

To summarize, early identification of intraoperative complications and their effective management are crucial to improving post-cataract surgery outcomes. The surgeon should adapt their approach as needed to optimize damage control. The objective in all cases should be to provide the best possible ophthalmic surgical care for the highest possible visual outcomes. Thus, it is the responsibility of the primary surgeon to diagnose the mishap in a timely manner and refer the patient immediately, while the onus of prompt and effective intervention with the best possible techniques, skills, and tools lies with the second surgeon. It is essential to support our fellow colleagues and work in coordination to enhance final visual outcomes in cases of abandoned cataract surgeries.

## Conclusions

Abandoned cataract surgeries and surgery-related complications are common causes of reduced visual outcomes. These challenges typically arise from inadequate preoperative evaluations and planning, leading to less favorable results. This study aims to discuss the spectrum of cases in which intraoperative mishaps led to the abandonment of surgery, with patients later being referred to a tertiary eye center for management. However, timely referral and intervention played a crucial role in the optimal management of these cases.

## Appendices

**Table 1:** Details of various cases with abandoned cataract surgery included in the study RE: right eye, LE: left eye, DMD: Descemet’s membrane detachment, IOL: intraocular lens, PC-IOL: posterior chamber intraocular lens, AC-IOL: anterior chamber intraocular lens, AS-OCT: anterior segment optical coherence tomography, DM: diabetes mellitus, HTN: hypertension, PEX: pseudo-exfoliation syndrome, HMSC: hypermature cataract, ICCE: intracapsular cataract extraction, DMEK: Descemet’s membrane endothelial keratoplasty, HMCF: hand movements close to face, Mi-OCT: microscope-integrated optical coherence tomography, DALK: deep anterior lamellar keratoplasty, AC: anterior chamber, PC: posterior capsule, PCR: posterior capsular rent, VAO: visual axis opacification, IA: irrigation-aspiration, UCVA: uncorrected visual acuity, SICS: small incision cataract surgery, AV: anterior vitrectomy, SFIOL: scleral fixated Intraocular lens

S. no.	Eye	Age/gender	Diagnosis	Step at which surgery abandoned	Time from primary surgery to presentation	Findings on presentation	Systemic risk factors	Ocular risk factors	Second surgery	Postoperative outcome
1.	LE	53/F	Post-cataract surgery corneal edema with DMD	After IOL insertion and visco - corneal edema	2 days (48 hours)	Corneal edema	Nil	Nil	Intracameral air	Reattachment of Descemet’s membrane, clear cornea
2.	RE	54/M	Post-cataract surgery DMD with focal corneal edema with honeycomb epitheliopathy	Superior Incision made and hydrated AS-OCT showed DMD	1 month	Honeycomb epitheliopathy (patient additionally on Rho kinase inhibitors)	DM	No	First stage, intracameral air, 2^nd^ stage cataract surgery	Descemet’s membrane re-attached, epithelial edema resolved
3.	RE	47/M	PEX with iridoschisis	Temporal Incision made	1 month	4 mm pupil, atonic, fibrillary white deposits, loss of pupillary ruff	DM	PEX iridoschisis, poor dilation	Phaco + PCIOL	Cornea clear and PC-IOL stable
4.	LE	87/M	HMSC with PEX and subluxation	After incision	3 days	Incisions made PEX, subluxation HMSC	DM	PEX subluxation	ICCE + AC-IOL	Cornea clear and AC-IOL stable
5.	LE	55/M	Abandoned cataract surgery with secondary glaucoma	After incision and intracameral injection of drugs	7 days	Superior PAS, superior sutured incision	Nil	No	Phacoemulsification + intraocular lens (temporal incision) (acetazolamide cover)	Clear cornea, stable IOL IOP controlled on single drug (timolol)
6.	LE	52/M	Pseudophakia with residual cortical sheet	IOL inserted - visco (PCR documented)	2 weeks	PCIOL with retrolental white lens matter VAO, opaque	Nil	Nil	Bimanual IA of the retrolental residual cortical sheet, PC intact	Good gain in vision UCVA 6/12
7.	RE	60/M	Pseudophakia with residual cortex	IOL inserted with retained cortical matter	2 weeks	PCIOL with retrolental white lens matter VAO, hazy	HTN	No	Bimanual IA of retrolental residual cortex	Improvement in vision, UCVA 6/9
8.	RE	55/M	Post cataract surgery corneal edema DMD	After IOL insertion and visco – hazy cornea	6 hours	Corneal edema, large wound (Seidel’s positive)	Nil	Nil	Auto DMEK	From HMCF to 6/6, endothelial cells viable result in clear cornea, vital role of Mi-OCT
9.	RE	74/F	Aphakia with wound leak with vitreous in AC with DMD with corneal edema	Nucleus delivery	4 days	Superior SICS wound open, vitreous touching endothelium, corneal edema DMD	DM	Diabetic HMSC documented outside	AV- SFIOL, wound closure, intracameral air	Postoperatively okay, corneal edema resolved over 1-month UCVA 6./24
10.	RE	58/M	Aphakia with vitreous prolapse through wound with DMD	Nucleus delivery	3 days	Superior SICS wound, vitreous strand outside the wound, corneal edema	Nil	HMSC with subluxation documented	AV-SFIOL, wound closure, intracameral air	Postoperatively okay, UCVA 6/12
11.	LE	30/M	Brittle cornea syndrome with corneal perforation	4 mm entry wound	2 hours	Flat AC, intumescent cataract, wound open and big	Nil	Other eye phthisis, d/t trivial trauma	Cataract surgery + DALK	AC formed PCIOL stable, graft-host well apposed
